# 肺癌疫苗研究进展

**DOI:** 10.3779/j.issn.1009-3419.2023.106.19

**Published:** 2023-09-20

**Authors:** Hao FAN, Xiangwei GE, Xin ZHOU, Yao LI, An WANG, Yi HU

**Affiliations:** ^1^100853 北京，中国人民解放军医学院; ^1^Chinese PLA Medical School, Beijing 100853, China; ^2^100071 北京，中国人民解放军总医院第五医学中心肿瘤医学部; ^2^Department of Oncology, the Fifth Medical Center, Chinese PLA General Hospital, Beijing 100071, China

**Keywords:** 肿瘤疫苗, 新抗原, 肺肿瘤, 第二代测序技术, Cancer vaccine, New antigen, Lung neoplasms, Next-generation sequencing

## Abstract

随着医学技术的发展，肿瘤疫苗作为一种新型精准免疫治疗手段在临床应用中逐渐受到重视。在全球新型冠状病毒（corona virus disease 2019, COVID-19）疫情爆发的背景下，疫苗技术的研发得到了进一步的发展。根据其抗原种类的不同，肿瘤疫苗可分为全细胞疫苗、多肽疫苗、信使核糖核酸（messenger ribonucleic acid, mRNA）疫苗、重组病毒疫苗等。尽管已经有一些肿瘤疫苗上市并取得了一定疗效，但过去一段时间肿瘤疫苗在临床试验中的结果仍不尽如人意，随着第二代测序技术（next-generation sequencing, NGS）的成熟和生物信息学的不断发展，使得肿瘤亚群发展的全过程动态跟踪成为了现实，为个性化定制以新抗原为核心的治疗性肿瘤疫苗打下了坚实的基础。本文回顾了近年来不同种类肿瘤疫苗的发展情况，以肺癌举例总结了肿瘤疫苗在临床应用中的发展成果，并对未来以新抗原为核心的肿瘤疫苗开发进行了展望。

## Abstract (en)

With the development of medical technology, tumor vaccines as a novel precise immunotherapy approach have gradually received attention in clinical applications. Against the backdrop of the global corona virus disease 2019 (COVID-19) outbreak, vaccine technology has further advanced. Depending on the types of antigens, tumor vaccines can be divided into whole-cell vaccines, peptide vaccines, messenger ribonucleic acid (mRNA) vaccines, recombinant virus vaccines, etc. Although some tumor vaccines have been marketed and achieved certain therapeutic effects, the results of tumor vaccines in clinical trials have been unsatisfactory in the past period. With the maturation of next-generation sequencing (NGS) technology and the continuous development of bioinformatics, dynamic monitoring of the entire process of tumor subpopulation development has become a reality, which has laid a solid foundation for personalized, neoantigen-centered therapeutic tumor vaccines. This article reviews the recent developments of tumor vaccines of different types, starts with lung cancer and summarizes the achievements of tumor vaccines in clinical applications, and provides an outlook for the future development of antigen-centered tumor vaccines.

肿瘤疫苗是近年来被广大学者广泛讨论的肿瘤治疗方法，其治疗原理是将肿瘤抗原以某种方式导入患者体内，激发或增强患者自身免疫系统，诱导机体产生长期的体液和细胞免疫应答，从而杀伤肿瘤细胞，作用机制如图1。肿瘤疫苗能够以肿瘤特异性抗原（tumor specific antigen, TSA）或肿瘤相关性抗原（tumor-associated antigen, TAA）为靶点，激活免疫系统，有效针对实体瘤或血液系统肿瘤^[1]^。由于肿瘤早期转移和微小病灶的特性，局部根治性治疗手段往往很难彻底治愈肿瘤，为了实现理想的治疗效果，疫苗刺激的免疫反应需要杀死全身的隐匿性病灶。自2010年美国食品药品监督管理局（Food and Drug Administration, FDA）批准人类历史上第一个前列腺癌治疗性疫苗-Provenge以来，全球开展的肿瘤疫苗临床研究总体呈上升的趋势，如图2。此外，肺癌、胃癌、前列腺癌等多种实体瘤的治疗性疫苗也已进入了临床试验，取得了一些积极的结果，部分已经获批于临床应用^[2⇓-4]^。但由于抗原制备困难、免疫原性低下、无法持久激活免疫系统等原因导致治疗性肿瘤疫苗的疗效与传统治疗方式相比不能尽如人意，目前仍存在一些问题需要进一步解决。例如，肿瘤抗原的多样性和复杂性使得疫苗设计和制备较为困难，且其治疗效果的评价标准尚不明确等^[5⇓-7]^。因此，肿瘤疫苗是值得深入研究的一个课题，未来需要进一步完善肿瘤疫苗的制备技术，并建立相应的评价标准，推动其在临床应用中的发展。本文将对最新肿瘤疫苗的临床研究进展及研发中遇到的困难和难点作一回顾性总结，探索未来肿瘤疫苗的发展方向。

**图1:**
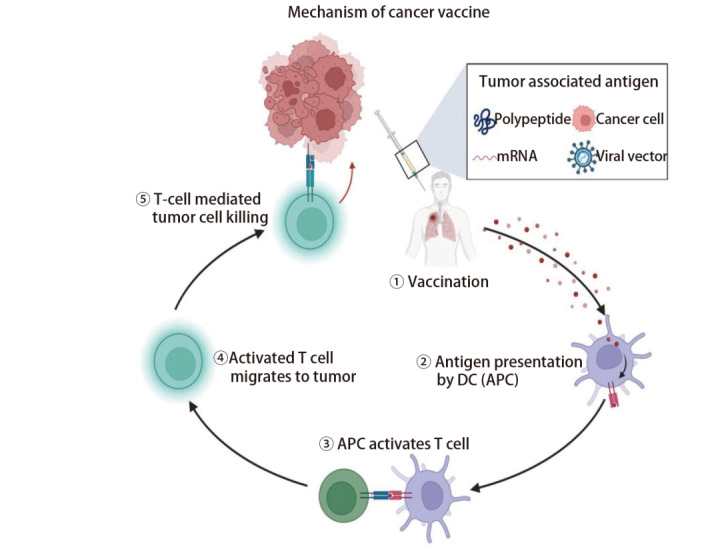
肿瘤疫苗作用机制

**图2:**
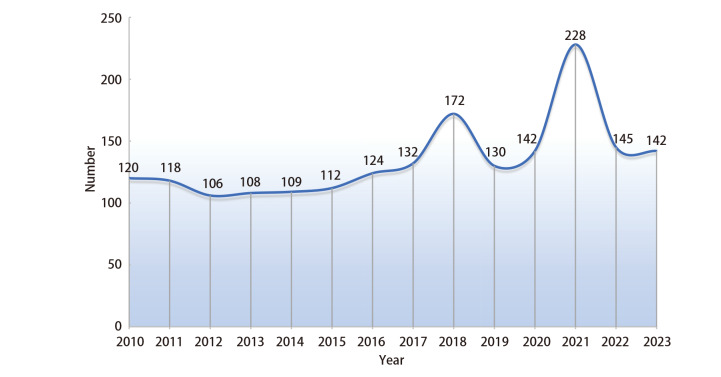
2010年以来肿瘤疫苗临床研究开展数量

## 1 肿瘤疫苗分类及治疗原理

根据FDA标准^[8]^，目前肿瘤疫苗按抗原类型分类可分为多肽疫苗、信使核糖核酸（messenger ribonucleic acid, mRNA）疫苗、全细胞疫苗、重组病毒疫苗等。利用肿瘤抗原制备肿瘤疫苗时一般与细胞因子或佐剂合用以激活更强的免疫反应，另外肿瘤疫苗的不同给药途径也可能造成疗效的差异，但可以肯定的是，已经有很多肿瘤疫苗在患者体内可以检测出有意义的免疫应答^[9,10]^。

### 1.1 多肽疫苗

多肽疫苗是利用已知病原体的某段抗原表位的氨基酸序列，通过化学合成方式人工合成短肽作为抗原诱发机体免疫应答，是目前临床研究最多的疫苗品种。由于多肽疫苗仅包含病原体的识别序列，可以诱发免疫应答但不致病，所以多肽疫苗技术广泛应用于抗结核和抗人类免疫缺陷病毒（human immunodeficiency virus, HIV）等多种病毒疫苗。多肽疫苗具有安全性高、可大规模合成、易于纯化和应用等特点。遗憾的是，由于其细胞摄取率低、快速清除率高、稳定性差等缺点，临床试验效果往往较为局限。多肽疫苗往往需要与免疫佐剂联合，或者通过病毒、树突状细胞（dendritic cell, DC）等合适的载体递送至免疫反应细胞来增强T细胞的免疫效应。

### 1.2 mRNA疫苗

mRNA疫苗是将编码外源性抗原的mRNA片段导入人体细胞内，由自体细胞的蛋白合成机制合成外源性抗原后引起全身免疫反应^[11,12]^。20世纪90年代，科学家Wolff等^[13]^利用小鼠实验，发现RNA/脱氧核糖核酸（deoxyribo nucleic acid, DNA）直接注射入体内可以导致相关蛋白表达显著增加，相关蛋白表达时间在2个月以上，为RNA疫苗打下了理论基础。mRNA疫苗的优点是可以同时呈递多种抗原，覆盖多种肿瘤特异性抗原（tumor-specific antigen, TSA）表位，与基于短肽疫苗容易受到主要组织相容性复合体（major histocompatibility complex, MHC）的限制不同，mRNA疫苗可以编码全长肿瘤抗原，允许抗原呈递细胞（antigen-presenting cell, APC）将多个抗原表位同时或交叉呈递，产生共刺激信号发挥佐剂作用，进一步激发T细胞效应^[14⇓⇓-17]^。此外，由于RNA无法整合至基因组中，避免了插入突变的可能，进一步提高了应用的安全性。然而，RNA疫苗也有一定的缺点，包括RNA稳定性较差、裸RNA摄取率很低、容易被降解等。所以，开发有效的RNA递送体、高效地将RNA穿透磷脂双分子层到达细胞质、有效提高免疫应答效应是疫苗疗效的重要保障^[18⇓-20]^。

### 1.3 其他类型疫苗

其他类型的肿瘤疫苗还包括肿瘤全细胞疫苗、DC疫苗、病毒载体疫苗等。全细胞疫苗以APC为靶点，将经过处理的肿瘤细胞制备为抗原，纯化后注射入体内引起机体免疫反应，或在离体条件下利用抗原先行激活APC，再将APC输入体内激活T细胞，后者一般采用体内抗原呈递活性最高的DC作为中介，也称为DC疫苗^[21⇓-23]^。全细胞疫苗不仅需要合适的佐剂来增强体内的免疫反应，提纯TSA或TAA的技术也同样重要，第二代测序技术（next-generation sequencing, NGS）和生物信息学工具可以让我们更好地了解到肿瘤亚群之间的关系，对整个突变过程的每个参数进行大规模分析，对于检测肿瘤亚群的突变、预测潜在表位、找到特异性新抗原有巨大的帮助^[24]^。新抗原一般在正常组织中不表达，仅在肿瘤组织中特异性表达，包括致瘤基因突变产生的突变蛋白和致瘤病毒整合进宿主基因产生的抗原等，新抗原不仅具有肿瘤特异性，还具有强免疫原性，新抗原强大的免疫原性可以帮助免疫细胞特异性杀伤肿瘤组织，比如Sebastian等^[25]^使用NGS技术对癌细胞系的人类白细胞抗原（human leukocyte antigen, HLA）进行转录组测序，并映射到HLA表型中，建立了每个细胞系中抗原突变的目录，为肿瘤疫苗的开发做出了贡献。病毒载体疫苗是将TSA或TAA插入载体病毒或细菌质粒，由重组微生物表达肿瘤抗原引起或增强机体免疫反应^[26,27]^。而具有溶瘤特性、经基因工程改造后的肿瘤靶向溶瘤病毒则是属于原位疫苗的类型，原位疫苗是在体内直接通过放疗、注射溶瘤病毒等方式激活APC，将肿瘤微环境诱导为富含效应细胞的免疫刺激环境，产生整体和长效的抗肿瘤反应，原位疫苗的关键步骤是诱导肿瘤细胞死亡、APC的募集和效应细胞的激活^[28,29]^。重组基因疫苗目前主要应用于癌症预防方面，比如重组人乳头瘤病毒（human papilloma virus, HPV）疫苗就是预防宫颈癌的有效武器。重组基因疫苗的优点是免疫系统已经建立了针对病毒的高效、强大、持久的免疫反应，通过病毒病原体相关的分子模式识别受体（pattern recognition receptor, PRR）来促进APC的激活。而缺点是体内强大的记忆性抗病毒免疫反应容易中和病毒抗原，从而降低了重复接种的可能。

## 2 肿瘤疫苗在临床中的应用价值

### 2.1 肿瘤疫苗在肺癌中的应用

肺癌作为世界上发病率位居第二位、死亡率位居第一位的癌症，也是癌症中免疫治疗和靶向治疗研究最为深入的病种。以肺癌举例，现阶段治疗性肿瘤疫苗的临床研究往往选择与常规治疗方式联合应用的治疗方案。Ott等^[30]^的研究表明肿瘤疫苗与程序性死亡受体1（programmed cell death 1, PD-1）药物纳武利尤单抗联合治疗，可以显著提高肿瘤疫苗的治疗效果，降低不良反应发生率，疫苗诱导的T细胞会随着时间的推移而持续存在，显示出细胞毒性潜力，并可以迁移到肿瘤微环境中杀伤肿瘤细胞；Gray等^[31]^在疫苗联合T细胞趋化因子CCL21（CC chemokine ligand 21）在晚期肺腺癌中的疗效的研究表明，免疫调节因子能够影响人体的免疫系统，从而增强肿瘤疫苗的疗效。肿瘤疫苗的递送方式和靶向器官在动物实验中也取得了一些进展，Chen等^[32]^认为将mRNA疫苗以脂质纳米颗粒的形式靶向输送到淋巴结内可以提高疗效，与PD-1药物联合治疗在黑色素瘤小鼠模型中完全缓解（complete response, CR）率达到了40%，并且表现出了长期免疫应答，CR的小鼠相比于未接种疫苗的小鼠，再次接种肿瘤组织后没有观察到肺部转移结节的发生。由于每个患者的癌症类型和免疫系统状态都不同，针对每个患者制定个性化的治疗方案更有指导意义，肿瘤疫苗的个体化诊疗也变得愈加重要。Fang等^[33]^分析了接受多肽疫苗治疗的非小细胞肺癌（non-small cell lung cancer, NSCLC）患者的T细胞受体（T cell receptor, TCR），开发了一个高通量的TCR分析系统，可以为接受免疫疗法的各种类型的患者提供详细的T细胞免疫反应信息，从而更好地了解肿瘤的发病机制和演变过程；Ding等^[34]^采集了复发的晚期肺癌患者血液和肿瘤标本进行了完整外显子组测序以找到体细胞非同义突变基因，此外还对肿瘤样本进行了RNA测序（RNA-sequencing, RNA-seq）以确认突变状态和确定可能的新抗原，使用个性化的新抗原合成多肽后在体外冲击自体DC制备成的疫苗成功激活了体内的T细胞，并产生了肿瘤杀伤效应。

### 2.2 肿瘤疫苗的安全性和有效性

大部分实体瘤早期临床研究仍然主要关注的是注射疫苗安全性和特异性免疫应答率，双臂试验中采用的治疗方式为疫苗治疗组对比安慰剂组居多，患者对疫苗没有产生明显的高级别不良反应，最常见的是注射部位的局部反应^[35⇓⇓-38]^。除了安全性，治疗周期和接种剂量也获得了不少研究者的关注，在一项尚未完成的队列^[39]^中接受高剂量DC疫苗治疗的患者总生存期（overall survival, OS）要明显优于低剂量DC疫苗的患者（P<0.0038）；Vreeland等^[40]^在2018年发起了一项研究，确定了减毒多肽疫苗的疗效和最佳治疗次序、周期；Brunsvig等^[41]^报道的研究确定了一种端粒酶逆转录酶疫苗在700 µg治疗剂量下对NSCLC治疗效果最好。产生疫苗特异性抗体的患者预后一般要好于预期，部分学者还提出免疫细胞的活性和持续时间的增加与临床获益相关，但整体人群临床最终结局OS和无进展生存期（progression-free survival, PFS）结果较少^[42⇓-44]^，这可能是由于疫苗制备技术不完善、肿瘤抗原预测准确性不高、MHC与T细胞亲和力较低、免疫应答细胞无法大规模杀伤肿瘤细胞所致，疫苗的临床效用转化为OS和PFS的提升仍然是一项艰巨的任务。在多种实体瘤的联合治疗方面，与PD-1药物历史治疗数据对比，联合治疗效果优于单用免疫治疗，也不会引起额外的免疫相关不良事件，这可能是联合治疗方案的优势^[30]^ 。一项针对乳腺癌治疗的研究^[45]^指出三阴性乳腺癌亚组可能对肿瘤疫苗获益更高，作者认为可能是由于疫苗与单克隆抗体产生了免疫协同作用；一项与传统化疗联合的研究^[46]^认为肿瘤疫苗可以提高T细胞功能，加强传统化疗方案的疗效。Kjeldsen等^[47]^的小样本量（n=15）研究指出，与晚期不可切除的NSCLC患者5年OS率小于5%的历史数据对比，一种多肽疫苗治疗中晚期NSCLC患者中位PFS为8.5个月，6年OS率为20%，并且有2例患者在没有接受其他抗肿瘤治疗的情况下在6年后仍有持续的临床反应，还有一些PFS和OS获益的小样本研究^[48]^需要扩大样本量进行下一阶段的临床试验。

### 2.3 肺癌相关临床试验结果

截至2023年9月，全球共有2936项与肿瘤疫苗有关的临床试验，以经济发达的美国和西欧地区为主，肺癌相关肿瘤疫苗临床试验有415项（数据来源：ClinicalTrials.gov）。一项2022年针对已注册的全球NSCLC治疗性疫苗临床试验特点的研究^[49]^指出，目前治疗性肿瘤疫苗的研究方向以多肽类疫苗为主（41.88%），研究对象主要为III或IV期患者（26.5%），大部分为单臂试验（52.14%），以小样本量为主（50人以下的试验占比56.41%）。这可能是由于多肽疫苗制备容易，副反应小，注射方式简单，可行性高，晚期患者也是免疫治疗的主要针对人群，而单臂小样本量试验可以降低获得试验结果的成本，尽管队列入组人数不多，但还是为免疫联合治疗方案提供了有利的依据。在近五年已发表的与肺癌相关的临床试验中（表1）可以观察到患者不良反应较少，对疫苗较敏感的实体瘤包括黑色素瘤、前列腺癌、多发性骨髓瘤、肾癌等，联合治疗方案也产生了一些生存获益^[55,56]^，但肿瘤疫苗在最终III期临床试验中治疗效果表现往往不及预期。黑色素瘤相关抗原3（melanoma antigen family A3, MAGE-A3）是肿瘤组织表达最多的肿瘤-睾丸抗原之一，具有严格的组织特异性，除睾丸和胎盘组织外在正常组织和细胞中不表达，但在30%-50%的NSCLC中表达，其中鳞癌的比例相对更高。Vansteenkiste等^[57]^针对MAGE-A3阳性的NSCLC患者进行了一项III期临床试验MAGRIT，MAGRIT是一项针对肿瘤特异性抗原MAGE-A3开发出的免疫疗法，这是迄今为止最大的肿瘤疫苗临床试验，有超过14,000例患者接受了筛查，最终取得了阴性结果，目前在临床I、II期试验结果较为理想的肿瘤疫苗如L-BLP25、Belagenpumatucel-L、TG4010等在不同癌种的III期临床试验结果均为阴性^[58⇓⇓⇓⇓⇓⇓⇓⇓⇓-68]^。

**表1:** 近5年发表的肺癌相关有代表性的肿瘤疫苗临床试验（数据来源：Clinicaltrials.gov）

Trial ID	Phase	Interventional model	Vaccine type	Participant group/Arm	Enrollment	Masking	Condition
NCT01935154^[4]^	Phase II	Parallel assignment	Peptide vaccine	Vx-001 vaccine/Placebo	221	Quadruple	NSCLC
NCT02897765^[30]^	Phase Ib	Single group assignment	Peptide vaccine	NEO-PV-01+Nivolumab+ Adjuvant	34	Open lable	Solid tumor
NCT01433172^[31]^	Phase I/II	Parallel assignment	DC vaccine	Dose gradient vaccine+Vaccine/Vaccine with chemotactic factor	73	Open lable	Lung adenocarcinoma
NCT02956551^[33]^	Phase I	Single group assignment	DC vaccine	Cell therapy (DC vaccine)	20	Open lable	NSCLC
NCT00923312^[34]^	Phase I /II	Single group assignment	mRNA vaccine	Vaccine	46	Open lable	NSCLC
NCT02840994^[35]^	Phase I	Parallel assignment	Viral vector vaccine	Vaccine+Pembrolizumab/Nivolumab	24	Open lable	Advanced malignant solid tumor
NCT02140996^[36]^	Phase I	Single group assignment	Viral vector vaccine	Vaccine	24	Open lable	Recurrent or metastatic solid tumor
NCT02669719^[38]^	Phase II	Parallel assignment	DC Vaccine	Pemetrexed+Carboplatin +dendritic cells/None	70	Open lable	NSCLC
NCT01789099^[40]^	Phase I/II	Single group assignment	Peptide vaccine	Vaccine+granulocyte macrophage-colony stimulating factor (GM-CSF)	18	Open lable	NSCLC
NCT02122861^[41]^	Phase I	Single group assignment	DC vaccine	Vaccine	47	Open lable	Solid tumor expressed NY-ESO-1
NCT01219348^[46]^	Phase I	Single group assignment	Peptide vaccine	IDO peptide vaccination	14	Open lable	NSCLC
NCT03380871^[50]^	Phase I	Single group assignment	Peptide vaccine	NEO-PV-01/adjuvant+ Pembrolizumab+ chemotherapy	38	Open lable	NSCLC
NCT02808416^[51]^	Phase I	Single group assignment	DC vaccine	Personalized cellular vaccine	10	Open lable	Solid tumor with brain metastases
NCT00828009^[52]^	Phase II	Single group assignment	Peptide vaccine	Chemoradiation+ Bevacizumab+Tecemotide	70	Open lable	NSNSCLC
NCT00617409^[53]^	Phase II	Factorial assignment	DC vaccine	Second line chemotherapy+Standard of care/Vaccine/Vaccine and all-trans-retinoic acid	69	Open lable	SCLC
NCT04300244^[54]^	Phase II	Parallel assignment	Telomerase vaccine	Ipilimumab and Nivolumab+Vaccine/None	118	Open lable	Malignant mesothelioma

NSCLC: non-small cell lung cancer; NSNSCLC: non-squamous non-small cell lung cancer; SCLC: small cell lung cancer.

### 2.4 肿瘤疫苗临床试验瓶颈

2010年美国Dendreon公司开发的前列腺癌疫苗Sipuleucel-T（商品名Provenge）为前列腺癌患者的治疗带来了里程碑式的突破^[2]^，与安慰剂组相比，死亡风险相对降低了22%，风险比（hazard ratio, HR）为0.78，95%置信区间（confidence interval, CI）为0.61-0.98（P=0.03），中位生存期延长了4.1个月（Sipuleucel-T组为25.8个月，安慰剂组为21.7个月），试验组的36个月OS率为31.7%，高于安慰剂组的23.0%，最终被FDA批准上市，成为全球首个治疗性肿瘤疫苗。但肿瘤疫苗研究遇到的问题主要还是在于相比于化疗、靶向治疗等免疫治疗手段没有比较优势，仅有部分试验相较安慰剂组获得了生存获益，这可能是受限于实体瘤的异质性高、抗原开发难度大、疫苗制备技术不完善等。我们认为不同肿瘤疫苗面对的技术难点也略有不同，多肽疫苗的缺点是递送系统较弱，免疫原性低，可以重点开发合适的佐剂，并适当增加疫苗的接种次数提高多肽疫苗的递送能力；mRNA疫苗的主要应用障碍在于mRNA易降解，很难长时间保证RNA的有效性，研究的重点应放在RNA序列设计方面，Zhang等^[69]^认为可以通过增加mRNA的二级结构等方式延长mRNA的半衰期，并设计了一种机器学习算法使得设计出的疫苗表现出高度稳定和效力更强的特征；病毒载体疫苗的劣势在于大部分人已经对病毒载体产生了免疫力，导致腺病毒、痘病毒等常用的载体无法激发特异性免疫反应，免疫原性低下，我们认为病毒载体的构建的关键在于增强载体的构建，如何增强载体复制能力、提高外源序列表达是下一步研究的重点。

### 2.5 临床试验设计的问题与不足

我们注意到很多临床试验中发生疾病进展、治疗副反应、病情恶化的患者往往不能完成整个治疗周期或由于改变治疗意愿等原因而提前退出试验^[31,64]^，最终完成全部疫苗治疗周期的人数相比于试验设计时少。Patel等^[52]^发起的临床试验入组筛选标准严格，需完成同步放化疗并巩固化疗4个周期后病情评估为稳定或缓解的患者才有资格进入疫苗联合贝伐珠单抗的维持治疗评价队列，最终完成治疗评价的人数不到入组人群的一半，停止治疗的最常见原因是疾病进展（16%），其次是毒性并发症（15%），所以得到的结论可能出现选择偏倚，病情较轻的患者在最终结果统计方面可能占比更高，同时由于疫苗的治疗方式和原理与免疫制剂类似，非严格设计的双盲试验容易让研究者产生信息偏倚。

## 3 肿瘤疫苗发展展望

随着肿瘤发生机制研究的深入，临床上对肿瘤的发生发展有了更加深刻的理解，免疫治疗受到了更多临床医生的关注^[6]^。近十年的基础研究充分说明基于通用肿瘤靶点的免疫治疗可以展现可观的临床治疗效果，而肿瘤疫苗概念的提出正是基于自体免疫反应治疗肿瘤的有效方案，其设计原理为治疗实体瘤提供了广阔的研究空间。与手术、放疗等局部治疗手段不同，肿瘤疫苗的优势在于可以通过免疫系统精准杀伤影像不可见的隐匿性病灶，为根治晚期实体瘤提供了可能。虽然肿瘤疫苗的研究尚处于起步阶段，但是随着新一代分子测序技术的普及、人工智能算法改进和疫苗制备工程的进步，肿瘤疫苗的研究思路将会发生革命性改变^[68,70⇓-72]^。肿瘤疫苗需要攻克的关键难题是根据不同的肿瘤抗原制备个性化的免疫反应，抗原的选择是肿瘤疫苗设计最重要的部分，其次就是通过佐剂或制备工艺提高疫苗的免疫效力，最终需要最优化的剂量、周期、部位注射方案来实现治疗目的^[73]^。目前来看新抗原似乎是理想条件下抗原的最优解，在临床研究中已经崭露头角，新抗原具有强大的免疫效应，而且新抗原又是肿瘤细胞生长发育所必须的，基本不存在免疫逃逸的可能，寻找合适的新抗原并将其以一定的方式导入APC或组成免疫复合物是解决肿瘤疫苗治疗难题的重中之重^[68,70,74]^。随着肿瘤新抗原提纯技术不断发展、抗原提呈过程的研究不断深入，肿瘤疫苗的设计和制备将会不断优化，实现增强免疫应答、改善免疫微环境、避免免疫逃逸，为肿瘤治疗提供了新的可能。

Competing interests

The authors declare that they have no competing interests.

## References

[1] Shemesh CS, Hsu JC, Hosseini I, et al. Personalized cancer vaccines: clinical landscape, challenges, and opportunities. Mol Ther, 2021, 29(2): 555-570. doi: 10.1016/j.ymthe.2020.09.038 33038322 PMC7854282

[2] Kantoff PW, Higano CS, Shore ND, et al. Sipuleucel-T immunotherapy for castration-resistant prostate cancer. N Engl J Med, 2010, 363(5): 411-422. doi: 10.1056/NEJMoa1001294 20818862

[3] Madan RA, Karzai F, Donahue RN, et al. Clinical and immunologic impact of short-course enzalutamide alone and with immunotherapy in non-metastatic castration sensitive prostate cancer. J Immunother Cancer, 2021, 9(3): e001556. doi: 10.1136/jitc-2020-001556 33664086 PMC7934713

[4] Gridelli C, Ciuleanu T, Domine M, et al. Clinical activity of a htert (vx-001) cancer vaccine as post-chemotherapy maintenance immunotherapy in patients with stage IV non-small cell lung cancer: final results of a randomised phase 2 clinical trial. Br J Cancer, 2020, 122(10): 1461-1466. doi: 10.1038/s41416-020-0785-y 32210365 PMC7217860

[5] Saxena M, van der Burg SH, Melief CJM, et al. Therapeutic cancer vaccines. Nat Rev Cancer, 2021, 21(6): 360-378. doi: 10.1038/s41568-021-00346-0 33907315

[6] Liu J, Fu M, Wang M, et al. Cancer vaccines as promising immuno-therapeutics: platforms and current progress. J Hematol Oncol, 2022, 15(1): 28. doi: 10.1186/s13045-022-01247-x 35303904 PMC8931585

[7] Lin MJ, Svensson-Arvelund J, Lubitz GS, et al. Cancer vaccines: the next immunotherapy frontier. Nat Cancer, 2022, 3(8): 911-926. doi: 10.1038/s43018-022-00418-6 35999309

[8] U.S. Department of Health and Human Services Food and Drug Administration. Guidance for Industry Content and Format of Chemistry, Manufacturing and Controls Information and Establishment Description Information for a Vaccine or Related Product[Internet]. Rockville, 1999 Jan [updated 2019 Aug 21; cited 2023 Oct 5]. Available from: http://www.fda.gov/cber/guidelines.htm http://www.fda.gov/cber/guidelines.htm

[9] Sultan H, Kumai T, Nagato T, et al. The route of administration dictates the immunogenicity of peptide-based cancer vaccines in mice. Cancer Immunol Immunother, 2019, 68(3): 455-466. doi: 10.1007/s00262-018-02294-5 30604041 PMC6428613

[10] Finn OJ. The dawn of vaccines for cancer prevention. Nat Rev Immunol, 2018, 18(3): 183-194. doi: 10.1038/nri.2017.140 29279613

[11] Weissman D. mRNA transcript therapy. Expert Rev Vaccines, 2015, 14(2): 265-281. doi: 10.1586/14760584.2015.973859 25359562

[12] Thess A, Grund S, Mui BL, et al. Sequence-engineered mRNA without chemical nucleoside modifications enables an effective protein therapy in large animals. Mol Ther, 2015, 23(9): 1456-1464. doi: 10.1038/mt.2015.103 26050989 PMC4817881

[13] Wolff JA, Malone RW, Williams P, et al. Direct gene transfer into mouse muscle in vivo. Science, 1990, 247( <W>4949 Pt 1):1465-1468. doi: 10.1126/science.1690918 1690918

[14] Faghfuri E, Pourfarzi F, Faghfouri AH, et al. Recent developments of RNA-based vaccines in cancer immunotherapy. Expert Opin Biol Ther, 2021, 21(2): 201-218. doi: 10.1080/14712598.2020.1815704 32842798

[15] Miao L, Zhang Y, Huang L. mRNA vaccine for cancer immunotherapy. Mol Cancer, 2021, 20(1): 41. doi: 10.1186/s12943-021-01335-5 33632261 PMC7905014

[16] Linares-Fernández S, Lacroix C, Exposito JY, et al. Tailoring mRNA vaccine to balance innate/adaptive immune response. Trends Mol Med, 2020, 26(3): 311-323. doi: 10.1016/j.molmed.2019.10.002 31699497

[17] Abdelaziz MO, Ossmann S, Kaufmann AM, et al. Development of a human cytomegalovirus (HCMV)-based therapeutic cancer vaccine uncovers a previously unsuspected viral block of MHC class I antigen presentation. Front Immunol, 2019, 10: 1776. doi: 10.3389/fimmu.2019.01776 31417555 PMC6682651

[18] Wang Y, Zhang Z, Luo J, et al. mRNA vaccine: a potential therapeutic strategy. Mol Cancer, 2021, 20(1): 33. doi: 10.1186/s12943-021-01311-z 33593376 PMC7884263

[19] To KKW, Cho WCS. An overview of rational design of mRNA-based therapeutics and vaccines. Expert Opin Drug Discov, 2021, 16(11): 1307-1317. doi: 10.1080/17460441.2021.1935859 34058918

[20] Huang T, Peng L, Han Y, et al. Lipid nanoparticle-based mRNA vaccines in cancers: Current advances and future prospects. Front Immunol, 2022, 13: 922301. doi: 10.3389/fimmu.2022.922301 36090974 PMC9458914

[21] Lokhov PG, Balashova EE. Cellular cancer vaccines: an update on the development of vaccines generated from cell surface antigens. J Cancer, 2010, 1: 230-241. doi: 10.7150/jca.1.230 21151581 PMC3001283

[22] Yang YL, Wang DH, Liao L, et al. Long-term efficacy of dendritic cell vaccines combined with cytokine-induced killer cells in the treatment of metastatic renal cell carcinoma. Zhongguo Zhongliu Shengwu Zhiliao Zazhi, 2019, 26(6): 695-699.

[23] Kranz LM, Diken M, Haas H, et al. Systemic RNA delivery to dendritic cells exploits antiviral defence for cancer immunotherapy. Nature, 2016, 534(7607): 396-401. doi: 10.1038/nature18300 27281205

[24] Selvakumar SC, Preethi KA, Ross K, et al. CRISPR/Cas9 and next generation sequencing in the personalized treatment of cancer. Mol Cancer, 2022, 21(1): 83. doi: 10.1186/s12943-022-01565-1 35331236 PMC8944095

[25] Sebastian A, Migalska M, Biedrzycka A. AmpliSAS and AmpliHLA: web server tools for MHC typing of non-model species and human using NGS data. Methods Mol Biol, 2018, 1802: 249-273. doi: 10.1007/978-1-4939-8546-3_18 29858815

[26] Guo ZS, Lu B, Guo Z, et al. Vaccinia virus-mediated cancer immunotherapy: cancer vaccines and oncolytics. J Immunother Cancer, 2019, 7(1): 6. doi: 10.1186/s40425-018-0495-7 30626434 PMC6325819

[27] Raja J, Ludwig JM, Gettinger SN, et al. Oncolytic virus immunotherapy: future prospects for oncology. J Immunother Cancer, 2018, 6(1): 140. doi: 10.1186/s40425-018-0458-z 30514385 PMC6280382

[28] Lurje I, Werner W, Mohr R, et al. In situ vaccination as a strategy to modulate the immune microenvironment of hepatocellular carcinoma. Front Immunol, 2021, 12: 650486. doi: 10.3389/fimmu.2021.650486 34025657 PMC8137829

[29] Jahan N, Ghouse SM, Martuza RL, et al. In situ cancer vaccination and immunovirotherapy using oncolytic HSV. Viruses, 2021, 13(9): 1740. doi: 10.3390/v13091740 34578321 PMC8473045

[30] Ott PA, Hu-Lieskovan S, Chmielowski B, et al. A phase Ib trial of personalized neoantigen therapy plus anti-PD-1 in patients with advanced melanoma, non-small cell lung cancer, or bladder cancer. Cell, 2020, 183(2): 347-362.e24. doi: 10.1016/j.cell.2020.08.053 33064988

[31] Gray JE, Chiappori A, Williams CC, et al. A phase I/randomized phase II study of GM.CD40L vaccine in combination with CCL21 in patients with advanced lung adenocarcinoma. Cancer Immunol Immunother, 2018, 67(12): 1853-1862. doi: 10.1007/s00262-018-2236-7 30209589 PMC6244998

[32] Chen J, Ye Z, Huang C, et al. Lipid nanoparticle-mediated lymph node-targeting delivery of mRNA cancer vaccine elicits robust CD8^+^ T cell response. Proc Natl Acad Sci U S A, 2022, 119(34): e2207841119. doi: 10.1073/pnas.2207841119 35969778 PMC9407666

[33] Fang H, Yamaguchi R, Liu X, et al. Quantitative T cell repertoire analysis by deep cDNA sequencing of T cell receptor α and β chains using next-generation sequencing (NGS). OncoImmunology, 2015, 3(12): e968467. doi: 10.4161/21624011.2014.968467 25964866 PMC4368121

[34] Ding Z, Li Q, Zhang R, et al. Personalized neoantigen pulsed dendritic cell vaccine for advanced lung cancer. Signal Transduct Target Ther, 2021, 6(1): 26. doi: 10.1038/s41392-020-00448-5 33473101 PMC7817684

[35] Sebastian M, Schröder A, Scheel B, et al. A phase I/IIa study of the mRNA-based cancer immunotherapy CV9201 in patients with stage IIIB/IV non-small cell lung cancer. Cancer Immunol Immunother, 2019, 68(5): 799-812. doi: 10.1007/s00262-019-02315-x 30770959 PMC11028316

[36] Rajan A, Gray JE, Devarakonda S, et al. Phase 1 trial of CV301 in combination with anti-PD-1 therapy in nonsquamous non-small cell lung cancer. Int J Cancer, 2023, 152(3): 447-457. doi: 10.1002/ijc.34267 36054490 PMC10690498

[37] Tan TJ, Ang WXG, Wang WW, et al. A phase I study of an adenoviral vector delivering a MUC1/CD40-ligand fusion protein in patients with advanced adenocarcinoma. Nat Commun, 2022, 13(1): 6453. doi: 10.1038/s41467-022-33834-4 36307410 PMC9616917

[38] Zhang W, Lu X, Cui P, et al. Phase I/II clinical trial of a Wilms’ tumor 1-targeted dendritic cell vaccination-based immunotherapy in patients with advanced cancer. Cancer Immunol Immunother, 2019, 68(1): 121-130. doi: 10.1007/s00262-018-2257-2 30306202 PMC11028035

[39] Zhong R, Ling X, Cao S, et al. Safety and efficacy of dendritic cell-based immunotherapy (DCVAC/LuCa) combined with carboplatin/pemetrexed for patients with advanced non-squamous non-small-cell lung cancer without oncogenic drivers. ESMO Open, 2022, 7(1): 100334. doi: 10.1016/j.esmoop.2021.100334 34959168 PMC8718955

[40] Vreeland TJ, Litton JK, Qiao N, et al. Phase Ib trial of folate binding protein (FBP)-derived peptide vaccines, E39 and an attenuated version, E39’: An analysis of safety and immune response. Clin Immunol, 2018, 192: 6-13. doi: 10.1016/j.clim.2018.03.010 29574039 PMC5988975

[41] Brunsvig PF, Guren TK, Nyakas M, et al. Long-term outcomes of a phase I study with UV1, a second generation telomerase based vaccine, in patients with advanced non-small cell lung cancer. Front Immunol, 2020, 11: 572172. doi: 10.3389/fimmu.2020.572172 33324397 PMC7726017

[42] Somaiah N, Block MS, Kim JW, et al. First-in-class, first-in-human study evaluating LV305, a dendritic-cell tropic lentiviral vector, in sarcoma and other solid tumors expressing NY-ESO-1. Clin Cancer Res, 2019, 25(19): 5808-5817. doi: 10.1158/1078-0432.CCR-19-1025 31227504

[43] Hung JT, Chen IJ, Ueng SH, et al. The clinical relevance of humoral immune responses to Globo H-KLH vaccine adagloxad simolenin (OBI-822)/OBI-821 and expression of Globo H in metastatic breast cancer. J Immunother Cancer, 2022, 10(6): e004312. doi: 10.1136/jitc-2021-004312 PMC9226869 35732348

[44] Huang CS, Yu AL, Tseng LM, et al. Globo H-KLH vaccine adagloxad simolenin (OBI-822)/OBI-821 in patients with metastatic breast cancer: phase II randomized, placebo-controlled study. J Immunother Cancer, 2020, 8(2): e000342. doi: 10.1136/jitc-2019-000342 PMC7380846 32718986

[45] Chick RC, Clifton GT, Hale DF, et al. Subgroup analysis of nelipepimut-S plus GM-CSF combined with trastuzumab versus trastuzumab alone to prevent recurrences in patients with high-risk, HER2 low-expressing breast cancer. Clin Immunol, 2021, 225: 108679. doi: 10.1016/j.clim.2021.108679 33485895

[46] Bernal-Estévez DA, Ortíz Barbosa MA, Ortíz-Montero P, et al. Autologous dendritic cells in combination with chemotherapy restore responsiveness of T cells in breast cancer patients: A single-arm phase I/II trial. Front Immunol, 2021, 12: 669965. doi: 10.3389/fimmu.2021.669965 34489928 PMC8417880

[47] Kjeldsen JW, Iversen TZ, Engell-Noerregaard L, et al. Durable clinical responses and long-term follow-up of stage III-IV non-small cell lung cancer (NSCLC) patients treated with IDO peptide vaccine in a phase I study-a brief research report. Front Immunol, 2018, 9: 2145. doi: 10.3389/fimmu.2018.02145 30283461 PMC6157336

[48] Toh U, Sakurai S, Saku S, et al. Early phase II study of mixed 19‐peptide vaccine monotherapy for refractory triple‐negative breast cancer. Cancer Sci, 2020, 111(8): 2760-2769. doi: 10.1111/cas.14510 32495455 PMC7419019

[49] Gu W, Xu Y, Chen X, et al. Characteristics of clinical trials for non-small cell lung cancer therapeutic vaccines registered on ClinicalTrials. gov. Front Immunol, 2022, 13: 936667. doi: 10.3389/fimmu.2022.936667 36341464 PMC9627174

[50] Awad MM, Govindan R, Balogh KN, et al. Personalized neoantigen vaccine NEO-PV-01 with chemotherapy and anti-PD-1 as first-line treatment for non-squamous non-small cell lung cancer. Cancer Cell, 2022, 40(9): 1010-1026.e11. doi: 10.1016/j.ccell.2022.08.003 36027916

[51] Anderson KS, Erick TK, Chen M, et al. The feasibility of using an autologous GM-CSF-secreting breast cancer vaccine to induce immunity in patients with stage II-III and metastatic breast cancers. Breast Cancer Res Treat, 2022, 194(1): 65-78. doi: 10.1007/s10549-022-06562-y 35482127 PMC9046531

[52] Patel JD, Lee JW, Carbone DP, et al. Phase II study of immunotherapy with Tecemotide and Bevacizumab after chemoradiation in patients with unresectable stage III non-squamous non-small cell lung cancer (NS-NSCLC): A trial of the ECOG-ACRIN cancer research group (E6508). Clin Lung Cancer, 2020, 21(6): 520-526. doi: 10.1016/j.cllc.2020.06.007 32807654 PMC7606595

[53] Chiappori AA, Williams CC, Gray JE, et al. Randomized-controlled phase II trial of salvage chemotherapy after immunization with a TP53-transfected dendritic cell-based vaccine (Ad.p53-DC) in patients with recurrent small cell lung cancer. Cancer Immunol Immunother, 2019, 68(3): 517-527. doi: 10.1007/s00262-018-2287-9 30591959 PMC6426813

[54] Haakensen VD, Nowak AK, Ellingsen EB, et al. NIPU: a randomised, open-label, phase II study evaluating nivolumab and ipilimumab combined with UV1 vaccination as second line treatment in patients with malignant mesothelioma. J Transl Med, 2021, 19(1): 232. doi: 10.1186/s12967-021-02905-3 34059094 PMC8165504

[55] Kantoff PW, Schuetz TJ, Blumenstein BA, et al. Overall survival analysis of a phase II randomized controlled trial of a Poxviral-based PSA-targeted immunotherapy in metastatic castration-resistant prostate cancer. J Clin Oncol, 2010, 28(7): 1099-1105. doi: 10.1200/JCO.2009.25.0597 20100959 PMC2834462

[56] Arlen P M, Skarupa L, Pazdur M, et al. Clinical safety of a viral vector based prostate cancer vaccine strategy. J Urol, 2007, 178(4 Pt 1):1515-1520. doi: 10.1016/j.juro.2007.05.117 17707059

[57] Vansteenkiste JF, Cho BC, Vanakesa T, et al. Efficacy of the MAGE-A3 cancer immunotherapeutic as adjuvant therapy in patients with resected MAGE-A3-positive non-small-cell lung cancer (MAGRIT): a randomised, double-blind, placebo-controlled, phase 3 trial. Lancet Oncol, 2016, 17(6): 822-835. doi: 10.1016/S1470-2045(16)00099-1 27132212

[58] Sangha R, Butts C. L-BLP25: a peptide vaccine strategy in non-small cell lung cancer. Clin Cancer Res, 2007, 13(15 Pt 2):s4652-s4654. doi: 10.1158/1078-0432.CCR-07-0213 17671159

[59] Singer CF, Pfeiler G, Hubalek M, et al. Efficacy and safety of the therapeutic cancer vaccine tecemotide (L-BLP25) in early breast cancer: Results from a prospective, randomised, neoadjuvant phase II study (ABCSG 34). Eur J Cancer, 2020, 132: 43-52. doi: 10.1016/j.ejca.2020.03.018 32325419

[60] Butts C, Murray N, Maksymiuk A, et al. Randomized phase IIB trial of BLP 25 liposome vaccine in stage IIIB and IV non-small-cell lung cancer. J Clin Oncol, 2005, 23(27): 6674-6681. doi: 10.1200/JCO.2005.13.011 16170175

[61] Schimanski CC, Kasper S, Hegewisch-Becker S, et al. Adjuvant MUC vaccination with tecemotide after resection of colorectal liver metastases: a randomized, double-blind, placebo-controlled, multicenter AIO phase II trial (LICC). Oncoimmunology, 2020, 9(1): 1806680. doi: 10.1080/2162402X.2020.1806680 32923171 PMC7458621

[62] Giaccone G, Bazhenova LA, Nemunaitis J, et al. A phase III study of belagenpumatucel-L, an allogeneic tumour cell vaccine, as maintenance therapy for non-small cell lung cancer. Eur J Cancer, 2015, 51(16): 2321-2329. doi: 10.1016/j.ejca.2015.07.035 26283035

[63] Nemunaitis J, Nemunaitis M, Senzer N, et al. Phase II trial of Belagenpumatucel-L, a TGF-beta 2 antisense gene modified allogeneic tumor vaccine in advanced non small cell lung cancer (NSCLC) patients. Cancer Gene Ther, 2009, 16(8): 620-624. doi: 10.1038/cgt.2009.15 19287371

[64] Nemunaitis J, Dillman RO, Schwarzenberger PO, et al. Phase II study of belagenpumatucel-L, a transforming growth factor beta-2 antisense gene-modified allogeneic tumor cell vaccine in non-small-cell lung cancer. J Clin Oncol, 2006, 24(29): 4721-4730. doi: 10.1200/JCO.2005.05.5335 16966690

[65] Dreicer R, Stadler WM, Ahmann FR, et al. MVA-MUC1-IL 2 vaccine immunotherapy (TG4010) improves PSA doubling time in patients with prostate cancer with biochemical failure. Invest New Drugs, 2009, 27(4): 379-386. doi: 10.1007/s10637-008-9187-3 18931824

[66] Oudard S, Rixe O, Beuselinck B, et al. A phase II study of the cancer vaccine TG 4010 alone and in combination with cytokines in patients with metastatic renal clear-cell carcinoma: clinical and immunological findings. Cancer Immunol Immunother, 2011, 60(2): 261-271. doi: 10.1007/s00262-010-0935-9 21069322 PMC11029770

[67] Quoix E, Lena H, Losonczy G, et al. TG4010 immunotherapy and first-line chemotherapy for advanced non-small-cell lung cancer (TIME): results from the phase 2b part of a randomised, double-blind, placebo-controlled, phase 2b/3 trial. Lancet Oncol, 2016, 17(2): 212-223. doi: 10.1016/S1470-2045(15)00483-0 26727163

[68] Rodriguez PC, Popa X, Martínez O, et al. A phase III clinical trial of the epidermal growth factor vaccine CIMAvax-EGF as switch maintenance therapy in advanced non-small cell lung cancer patients. Clin Cancer Res, 2016, 22(15): 3782-3790. doi: 10.1158/1078-0432.CCR-15-0855 26927662

[69] Zhang H, Zhang L, Lin A, et al. Algorithm for optimized mRNA design improves stability and immunogenicity. Nature, 2023, 621(7978): 396-403. doi: 10.1038/s41586-023-06127-z 37130545 PMC10499610

[70] Kiyotani K, Toyoshima Y, Nakamura Y. Personalized immunotherapy in cancer precision medicine. Cancer Biol Med, 2021, 18(4): 955-965. doi: 10.20892/j.issn.2095-3941.2021.0032 34369137 PMC8610159

[71] Zhou C, Wei Z, Zhang Z, et al. pTuneos: prioritizing tumor neoantigens from next-generation sequencing data. Genome Med, 2019, 11(1): 67. doi: 10.1186/s13073-019-0679-x 31666118 PMC6822339

[72] Richters MM, Xia H, Campbell KM, et al. Best practices for bioinformatic characterization of neoantigens for clinical utility. Genome Med, 2019, 11(1): 56. doi: 10.1186/s13073-019-0666-2 31462330 PMC6714459

[73] Bowen WS, Svrivastava AK, Batra L, et al. Current challenges for cancer vaccine adjuvant development. Expert Rev Vaccines, 2018, 17(3): 207-215. doi: 10.1080/14760584.2018.1434000 29372660 PMC6093214

[74] Xie N, Shen G, Gao W, et al. Neoantigens: promising targets for cancer therapy. Signal Transduct Target Ther, 2023, 8(1): 9. doi: 10.1038/s41392-022-01270-x 36604431 PMC9816309

